# Microbial and Metabolomic Insights into Lactic Acid Bacteria Co-Inoculation for Dough-Stage Triticale Fermentation

**DOI:** 10.3390/microorganisms13081723

**Published:** 2025-07-23

**Authors:** Yujie Niu, Xiaoling Ma, Chuying Wang, Peng Zhang, Qicheng Lu, Rui Long, Yanyan Wu, Wenju Zhang

**Affiliations:** Animal Nutrition and Feed Science, College of Animal Science and Technology, Shihezi University, Shihezi 832000, China; niuyujie2025@163.com (Y.N.); ymn0522@163.com (X.M.); 18509932185@163.com (C.W.); 15297594947@163.com (P.Z.); 18299088259@163.com (Q.L.); l17365213918@sina.com (R.L.)

**Keywords:** triticale silage, lactic acid bacteria, bacterial diversity, metabolite profiles

## Abstract

Triticale (*Triticosecale Wittmack*) is a versatile forage crop valued for its high yield, balanced nutrition, and environmental adaptability. However, the dough-stage triricale has higher dry matter and starch content but lower water-soluble carbohydrate levels than earlier stages, posing fermentation challenges that may impair silage quality. This study aimed to investigate the effects of lactic acid bacteria inoculation on the fermentation quality, bacterial community, and metabolome of whole-plant triticale silage at the dough stage. Fresh triticale was ensiled for 30 days without or with an inoculant containing *Lactiplantibacillus plantarum* and *Streptococcus bovis*. Fermentation quality, bacterial succession, and metabolic profiles were analyzed at multiple time points. Inoculation significantly improved fermentation quality, characterized by a rapid pH drop, increased lactic acid production, and better preservation of fiber components. Microbial analysis revealed that inoculation successfully established *Lactobacillus* as the dominant genus while suppressing spoilage bacteria like *Enterobacter* and *Clostridium*. Metabolomic analysis on day 30 identified numerous differential metabolites, indicating that inoculation primarily altered pathways related to amino acid and purine metabolism. In conclusion, inoculating dough-stage triticale with this LAB combination effectively directs the fermentation trajectory. It enhances silage quality not only by optimizing organic acid profiles and microbial succession but also by modulating key metabolic pathways, ultimately leading to improved nutrient preservation.

## 1. Introduction

Triticale (*Triticosecale Wittmack*), a wheat-rye hybrid, combines wheat’s high yield with rye’s cold and drought tolerance [1]. Its high protein content and forage yield have driven expanded cultivation in Chinese regions lacking high-quality forage [2]. Harvesting at the dough-stage is often practiced to maximize dry matter (DM) accumulation and total digestible nutrient yield, due to starch deposition in the grain [3]. However, compared to the milk stage [4], dough-stage triticale has higher dry matter (DM) and starch content, which may hinder compaction and increase aerobic spoilage. Its higher starch-to-water-soluble carbohydrate (WSC) ratio can slow initial acidification if epiphytic lactic acid bacteria (LAB) inefficiently utilize these substrates [5].

Natural fermentation limitations arise from epiphytic microbial communities promoting undesirable bacterial growth, which reduces silage quality [6]. LAB additives are essential for silage fermentation, rapidly producing organic acids to lower pH and inhibit spoilage microbes [7]. Common silage inoculants include *Lactiplantibacillus plantarum*, *Lentilactobacillus buchneri*, *Pediococcus pentosaceus*, and *Enterococcus faecium* [8,9,10]. Among these, *L. plantarum* is widely used for its rapid acidification of silage via LA production. However, single-strain inoculation often yields suboptimal fermentation under diverse conditions, while multi-strain inoculation offers complementary benefits [11]. For example, co-inoculating *L. plantarum* and *L. buchneri* lowers pH, increases acetic acid (AA), and enhances aerobic stability [11]. Similarly, Li et al. [10] indicated that the synergistic effect of *L. plantarum* and *Bacillus coagulans* improved the fermentation quality of triticale silage.

*Streptococcus bovis*, a lactic acid-producing bacterium, has drawn attention in silage research for its proliferation rate, over 30% faster than other LABs (such as *Leuconostoc mesenteroides* and *Levilactobacillus brevis*) and breaks down starch into glucose and fermentable sugars, contributing to increased LA formation during ensiling [12,13,14]. Studies show that *S. bovis* inoculation in tropical grass silages lowers pH and ammonia nitrogen (AN), improves DM recovery [15], and reduces crude protein losses [16]. Our previous research indicated that *S. bovis* inoculation may be inadequate in complex fermentation environments, whereas co-inoculation with *L. plantarum* and *P. pentosaceus* improves silage quality by increasing LA, antioxidant capacity, and DM retention while lowering pH and AN [17].

High-throughput sequencing and metabolomics have enabled detailed exploration of microbial communities and metabolic changes in silage fermentation, revealing strong links between microbial communities and metabolite profiles [18,19]. However, despite the promising attributes of *S. bovis*, its specific role and impact on the fermentation microbiome and metabolome of dough-stage triticale silage, particularly the mechanistic details of its synergistic interactions when co-inoculated with well-established inoculants like *L. plantarum*, remain largely uninvestigated.

Therefore, this study aimed to investigate the individual and combined effects of *S. bovis* and *L. plantarum* on fermentation quality, bacterial community, and metabolite profile of dough-stage triticale silage. We hypothesize that co-inoculating *S. bovis* with *L. plantarum* synergistically enhances triticale silage fermentation, improving acid production and nutrient preservation compared to single inoculants. The findings from this research, utilizing 16S rRNA gene sequencing and untargeted metabolomics, are expected to clarify the mechanisms by which these LAB inoculants improve dough-stage triticale silage fermentation and provide a basis for developing more effective silage additives.

## 2. Materials and Methods

### 2.1. Silage Preparation

Triticale (*Triticosecale Wittmack*; Varitety: Shida No.1) was harvested on the 13th of June 2022 from the experimental farm of Shihezi University (Changji, Xinjiang, China). The fresh triticale was harvested at the dough maturity stage and subsequently sectioned into lengths of approximately 2 cm using a forage chopper. Four treatment groups were prepared: (1) control (CON), sprayed with sterile distilled water; (2) *S. bovis* inoculant (ST, accession: OQ812187); (3) *L. plantarum* inoculant (LP, Shandong Zhongke Jiayi Bioengineering Co., Ltd., Weifang, China); and (4) combined *S. bovis* + *L. plantarum* (LS,1:1). Each inoculant was applied at 10^6^ CFU/g FM, respectively. The inoculant was uniformly applied to the samples at a concentration of 10^6^ CFU/g FM by spraying. After thorough mixing, 1000 g of treated forage was packed into 2 L laboratory silos. An ensiling density of 500 kg/m^3^ was achieved through compaction and subsequent vacuum sealing. The silos were subsequently incubated at an ambient temperature of 22–26 °C to permit fermentation. Following 7 and 30 days of fermentation, the silos were unsealed for sample collection. Quintuplicate silos per treatment were prepared for each fermentation period. Upon opening, silage from each silo was thoroughly mixed. Collected subsamples were subjected to immediate analysis to determine fermentation quality and characterize the bacterial community structure. Additional subsamples from the 30-day silages were stored at −80 °C for metabolomic analysis.

### 2.2. Fermentation Characteristics Analyses

For the DM content, a 200 g subsample from each silo was dried at 65 °C for 48 h in a forced-air oven. For subsequent chemical analysis, the dried samples were milled to pass through a 1 mm sieve and then stored at −20 °C. The quantification of starch was performed using a commercial assay kit (Solarbio, Beijing, China). Crude protein (CP) content was determined by the Kjeldahl method according to AOAC procedures [20]. WSC content was measured by the anthrone colorimetric method [21]. The contents of acid detergent fiber (ADF) and neutral detergent fiber (NDF) were determined following the procedure outlined by Van Soest et al. [22]. To prepare silage extract for fermentation product analysis, an aqueous extract was prepared by macerating a 20 g silage sample in 180 mL of sterile distilled water at 4 °C for 24 h, after which the mixture was filtered through four layers of cheesecloth. The filtrate was used to measure pH value, LA, AA, propionic acid (PA), and butyric acid (BA), and AN. The pH was determined using a pH meter (WTW pH 3110, Xylem Inc. Munich, Germany). The organic acids were quantified by HPLC 1290 (Agilent Technologies, Inc., Santa Clara, CA, USA) equipped with a C18 column (150 mm × 4.6 mm; FMF-5559-EONU FLM Scientific Instruments Co., Ltd., Guangzhou, China). The analysis was performed under the following conditions: the mobile phase was Na_2_HPO_4_ (1 mM), delivered at a flow rate of 0.6 mL/min. The column temperature was maintained at 50 °C, and the injection volume was 20 µL. Detection was carried out using a Diode Array Detector (DAD) at a wavelength of 210 nm. The total run time for each sample was 25 min. The concentration of AN was measured using the phenol-hypochlorite colorimetric method [23]. Specifically, 1 mL of silage extract was mixed with 2 mL of a 5% (*w*/*v*) phenol solution containing 0.025% (*w*/*v*) sodium nitroprusside as a catalyst, followed by the addition of 2 mL of a 0.5% (*v*/*v*) sodium hypochlorite solution in a 0.125 M sodium borate buffer. The mixture was incubated in a 37 °C water bath for 30 min, and the absorbance was measured at 630 nm. A standard curve was generated using ammonium sulfate.

The viable counts of LAB, *yeasts*, *clostridium butyricum*, and *molds* in the fresh triticale were determined by plate culturing (pour-plate method) on appropriate agar media, following the procedures described by Chen et al. [24].

### 2.3. Bacterial Community Analysis

Total genomic DNA was isolated from silage samples employing the DNeasy PowerSoil Kit (QIAGEN, Netherlands) in accordance with the manufacturer’s protocol. The extracted DNA was stored at −20 °C until analysis. DNA concentration and purity were assessed using a NanoDrop ND-1000 spectrophotometer (Thermo Fisher Scientific, Waltham, MA, USA) and by 1% agarose gel electrophoresis, respectively. The V3–V4 region of the bacterial 16S rRNA gene was amplified by PCR using the primers 338F (‘5-ACTCCTACGGGAGGCAGCAG-3′) and 806R (‘5-GGACTACHVGGGTWTCTAAT-3′). Each 25 μL PCR reaction was composed of the following: 5 μL of Q5 Reaction Buffer, 5 μL of Q5 High GC Enhancer, 0.25 μL of Q5 DNA Polymerase, 2 μL of a dNTP mix (2.5 mM each), 1 μL of each primer (10 μM), 2 μL of template DNA, and 8.75 μL of nuclease-free water. The PCR amplicons were subjected to purification with Agencourt AMPure Beads (Beckman Coulter, Indianapolis, IN), followed by quantification using the PicoGreen dsDNA Assay Kit (Invitrogen, Carlsbad, CA, USA). Sequencing was performed on the Illumina MiSeq platform with a MiSeq Reagent Kit v3 (Shanghai Personal Biotechnology Co., Ltd., Shanghai, China). The resulting raw data were subsequently processed using the Quantitative Insights into Microbial Ecology (QIIME2, 2019.4) pipeline. For the bacterial community data, the Dada2 algorithm [25] was applied for the removal of primers, quality filtering, denoising, sequence splicing, and chimera elimination, resulting in the generation of Amplicon Sequence Variants (ASVs). The bacterial community composition was assessed at the phylum and genus levels by referencing the Silva database (Release 132, http://www.arb-silva.de. Accessed on 30 April 2024).

### 2.4. Metabolite Analysis

For each experimental treatment, five independent silage subsamples were submitted for untargeted metabolomic profiling at Shanghai Personal Biotechnology Co., Ltd. (Personalbio, Shanghai, China). Sample pretreatment and instrument conditions according to Huang et al. [26]. Briefly, A 100 mg aliquot of the powdered material was placed into a 2 mL microcentrifuge tube and combined with 400 µL of an ice-cold extraction solvent (acetonitrile:methanol, 1:1, *v*/*v*), which was fortified with 0.02 mg mL^−1^ L-2-chlorophenylalanine as an internal standard. Following a 30-s vortexing step, the samples were subjected to sonication for 30 min at 40 kHz and 5 °C, incubated at −20 °C for 30 min to precipitate proteins and particulates, and centrifuged (13,000× *g*, 4 °C, 15 min). The supernatant was subsequently evaporated to dryness under a gentle nitrogen stream, and the resultant residue was reconstituted in 100 µL of a 1:1 (*v*/*v*) acetonitrile:water solution. The reconstituted solution was sonicated for 5 min at 5 °C, centrifuged again (13,000× *g*, 4 °C, 10 min), and the final supernatant was transferred to glass vials for metabolites analysis.

Metabolite separation utilized an Agilent 1290 ultra-high-performance liquid chromatograph coupled to an Agilent 6460 triple-quadrupole mass spectrometer (UHPLC-MS/MS, Agilent Technologies, Santa Clara, CA, USA), equipped with an electrospray ionization source. Chromatography employed a Waters HSS T3 column (100 mm × 2.1 mm i.d., 1.8 µm). The injection volume was 3 µL; the column was maintained at 40 °C and eluted at 0.40 mL min^−1^. Mobile phase A consisted of 95% H_2_O/5% acetonitrile with 0.1% formic acid, whereas mobile phase B comprised 47.5% acetonitrile, 47.5% isopropanol, and 5% H_2_O with 0.1% formic acid. Mass spectra were acquired in both positive and negative ion modes over m/z 70–1050. Key source parameters were as follows: spray voltage ±3500 V, capillary temperature 325 °C, sheath gas 50 psi, auxiliary gas 13 psi, and auxiliary heater 425 °C. A stepped collision energy of 20, 40, and 60 eV was applied in a data-dependent acquisition (DDA) cycle. Resolving power was set to 60,000 (MS^1^) and 7500 (MS^2^). Raw data were imported into Progenesis QI (Waters Corp., Milford, CT, USA) for peak detection, alignment, and normalization. Partial least-squares discriminant analysis (PLS-DA) [27] was performed in R (v 4.2.2). Differential metabolites between each treatment and the control were identified based on a VIP score ≥ 1, fold change ≥ 2, and *p* < 0.05. Metabolite annotation and pathway mapping were conducted using the KEGG compound database (http://www.kegg.jp/kegg/compound. Accessed on 16 April 2024) and the KEGG pathway database (http://www.kegg.jp/kegg/pathway.html. Accessed on 16 April 2024), respectively.

### 2.5. Statistical Analysis

The impact of inoculants on bacterial populations and silage characteristics was statistically evaluated by one-way analysis of variance (ANOVA), conducted with IBM SPSS software version 22.0. Tukey’s test was used for post hoc multiple comparisons among treatment means. A *p*-value less than 0.05 was set as the threshold for statistical significance. Spearman rank correlation analysis was employed to explore the relationships between the predominant bacterial communities and the detected metabolites.

## 3. Results

The chemical composition and initial bacterial populations of fresh triticale before ensiling are presented in Table 1. The fresh material exhibited a pH of 6.23. The contents of WSC, starch, CP, NDF, and ADF were 72.5, 213, 85.7, 632, and 301 g/kg of DM, respectively. The initial epiphytic bacterial counts (log_10_ CFU/g FM) were 3.22 for LAB, 4.50 for yeasts, 4.34 for molds, and 7.66 for aerobic bacteria.

### 3.1. Silage Characteristics of Triticale Silage

**Figure 1:**
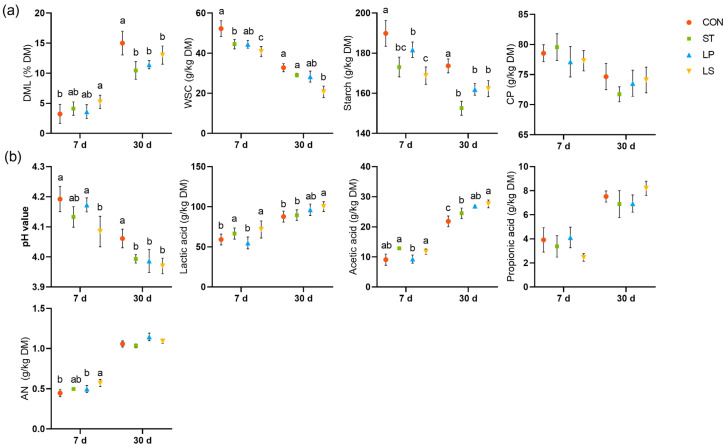
Effects of different inoculants on the chemical composition and fermentation characteristics of triticale silage at 7 and 30 days. (**a**), effects of different inoculants on the chemical composition of triticale silage at 7 and 30 days; (**b**), effects of different inoculants on fermentation characteristics of triticale silage at 7 and 30 days DML, dry matter loss; WSC, water-soluble carbohydrate; CP, crude protein; AN, ammonia nitrogen; CON, control group; ST, inoculation with *S. bovis*; LP, inoculation with *L. plantarum*; LS, co-inoculation with *S. bovis* and *L. plantarum*; a–c within the same ensiling day, values designated with different lowercase letters indicate statistically significant differences between treatments (*p* < 0.05).

The chemical composition, fermentation characteristics, and culturable microbial counts of triticale silage after 7 and 30 days are presented in Figure 1 and Figure S1, respectively. As shown in Figure 1a, dry matter loss (DML) was significantly lower in all inoculated treatments compared to CON at 30 days (*p* < 0.05). The pH, WSC, starch, and CP content decreased (*p* < 0.05), while LA, AA, PA, and AN concentrations increased across treatments over time (*p* < 0.05). Throughout ensiling, the CON group consistently exhibited higher WSC and starch content compared to the LS group (*p* < 0.05). After 7 days of ensiling, silages inoculated with ST and LS exhibited lower WSC content compared to CON (*p* < 0.05), and lower starch content compared to both CON and LP (*p* < 0.05). As shown in Figure 1b, ST and LS treatments displayed higher LA and AA concentrations compared to CON and LP (*p* < 0.05). By day 30, the LS treatment achieved the highest LA and AA concentrations. The AN concentration, while generally low across all treatments, was significantly higher in the LS group compared to CON and LP at 7 days of ensiling (*p* < 0.05). BA was not detected in all treatments.

The counts of culturable LAB in inoculated treatments tended to be higher than in CON (*p* < 0.05) at either 7 or 30 days (Figure S1). *Yeasts*, *molds*, and *Clostridium butyricum* were not detected in any treatments throughout the ensiling.

### 3.2. Analysis of Bacterial Communities

**Figure 2:**
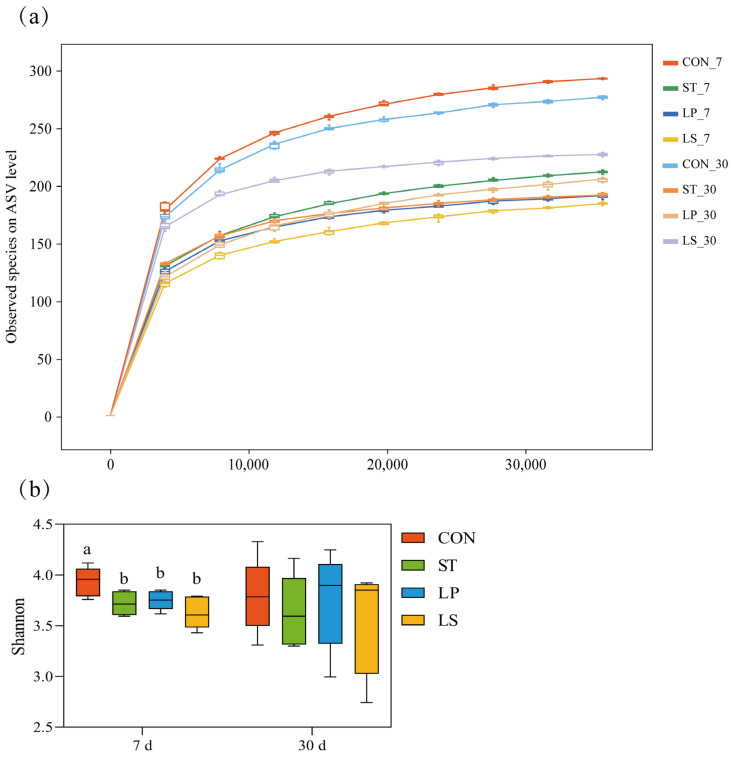
(**a**) Rarefaction curve showing the number of ASVs of triticale silage on different treatments and ensiling days. (**b**) Community diversity of triticale silage on different treatments and ensiling days. CON, control group; ST, inoculation with *S. bovis*; LP, inoculation with *L. plantarum*; LS, co-inoculation with *S. bovis* and *L. plantarum*; a,b values with different lowercase letters with the same ensiling day indicate significant differences among treatments (*p* < 0.05).

As shown by the rarefaction curves (Figure 2a), the sequencing depth was sufficient, with curves approaching a plateau for all samples. After 7 days of ensiling, the Shannon diversity index of the CON silage was significantly higher than those of the inoculated silages (*p* < 0.05), while there were no significant differences among the inoculated groups (Figure 2b). By day 30, Shannon indices did not differ significantly among treatments (*p* > 0.05).

**Figure 3:**
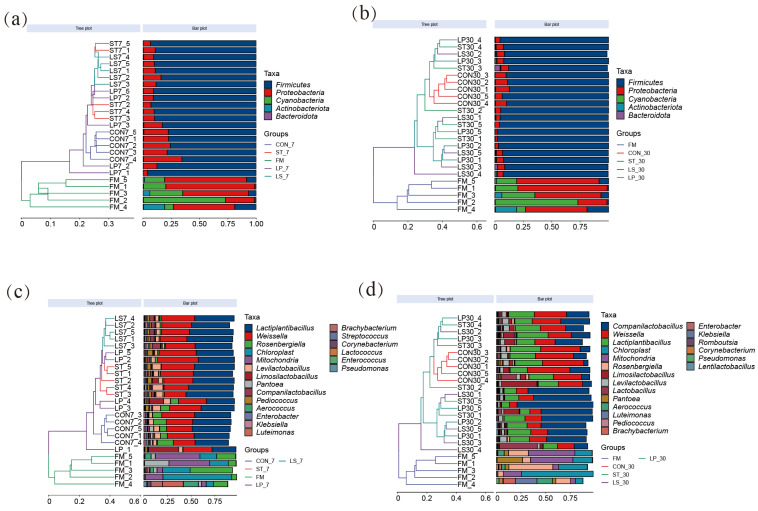
Hierarchical clustering and stacked bar plots of bacterial communities at phylum (**a**,**c**) and genus (**b**,**d**) levels on days 7 and 30. FM, fresh matter; CON, control group; ST, inoculation with *S. bovis*; LP, inoculation with *L. plantarum*; LS, co-inoculation with *S. bovis* and *L. plantarum*.

High-throughput 16S rRNA sequencing was performed on the fresh forage and silage samples, and the top 5 phyla and top 20 genera were analyzed. At day 7, fresh material formed a distinct cluster, while CON samples clustered separately from the LAB-inoculated samples (ST, LP, LS), which grouped closely together (Figure 3a,c). After 30 days, CON silage remained distinct, whereas the inoculated silages, while still separated from CON, showed less distinct separation among themselves (Figure 3b,d).

At the phylum level (Figure 3a,b; Table S1), at day 7 (Figure 3a), the CON silages had a significantly lower abundance of Firmicutes (74.94%) and a higher abundance of Proteobacteria (24.49%) compared to the inoculated silages (Firmicutes: 88.34–90.95%; Proteobacteria: 8.72–11.18%; *p* < 0.001 for both phyla). A similar trend, though less pronounced for Firmicutes (*p* = 0.076), was observed at day 30 (Figure 3b), where Proteobacteria remained significantly higher in CON (9.70%) compared to inoculated groups (4.21–5.41%; *p* = 0.001).

At the genus level (Figure 3c,d; Table S2), *Chloroplast* (29.31%) is the dominant genus in fresh samples, followed by *Mitochondria* (23.85%) and *Rosenbergiella* (18.58%). After 7 days of ensiling (Figure 3c), *Lactiplantibacillus* (33.34–44.48%) and *Weissella* (28.19–35.08%) were the dominant genera. Notably, ST and LS silages exhibited significantly higher relative abundances of *Lactiplantibacillus* compared to CON and LP (*p* < 0.05). All inoculated silages showed significantly lower abundances of potentially undesirable genera such as *Rosenbergiella*, *Enterobacter*, and *Pantoea* compared to CON (*p* < 0.05). ST and LS treatments also had a higher abundance of *Lactococcus* relative to CON (*p* < 0.05). After 30 days of ensiling (Figure 3d), a notable shift occurred: *Companilactobacillus* became the dominant genus in all inoculated silages (ST: 35.17%, LP: 42.15%, LS: 35.80%), while its abundance in CON was significantly lower (21.13%) (*p* = 0.041). Concurrently, the relative abundances of *Lactiplantibacillus* and *Weissella* decreased in all treatments compared to day 7. The CON silage maintained significantly higher abundances of *Enterobacter*, *Pantoea*, and *Klebsiella*, and lower abundances of *Companilactobacillus* and *Lactobacillus* compared to inoculated silages at day 30 (*p* < 0.05). The LS silage exhibited the highest relative abundance of *Lactobacillus* at day 30.

**Figure 4:**
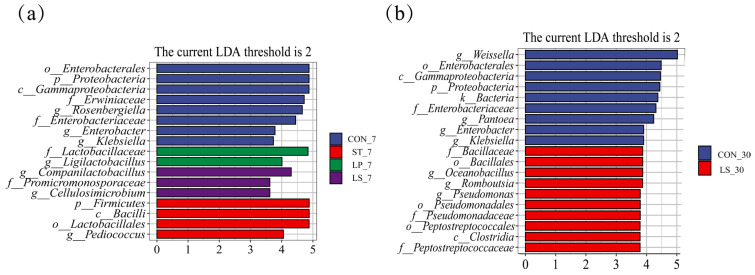
LEfSe analysis of triticale silage with different treatments for 7 (**a**) and 30 (**b**) days.

LEfSe analysis (LDA score > 2.0) further highlighted the distinctive genera in each treatment (Figure 4). At day 7, *Rosenbergiella*, *Enterobacter*, and *Klebsiella* were significantly enriched in CON. *Ligilactobacillus* was a biomarker for LP, *Pediococcus* for ST, and *Companilactobacillus* and *Cellulosimicrobium* for LS. By day 30, *Weissella*, *Pantoea*, *Enterobacter*, and *Klebsiella* remained enriched in CON. *Romboutsia* and *Pseudomonas* were identified as biomarkers for the LS (Figure 4b).

### 3.3. Analysis of Metabolite

**Figure 5:**
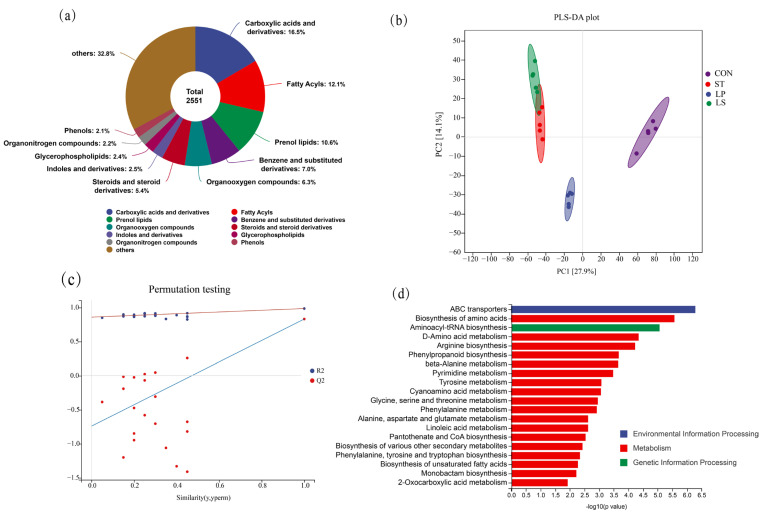
Metabolomic profiling and multivariate analysis of triticale silage after 30 days of ensiling; (**a**) chemical classification of the annotated metabolites in all treatments; (**b**) analysis of PLS-DA score plots with different treatments; (**c**) permutation test results for the PLS-DA model; (**d**) KEGG pathway enrichment analysis of differentially abundant metabolites; CON, control group; ST, inoculation with *S. bovis*; LP, inoculation with *L. plantarum*; LS, co-inoculation with *S. bovis* and *L. plantarum*.

Untargeted metabolomic analysis of day 30 silages identified 3590 metabolite features, of which 2551 were annotated against the KEGG database (Table S3). The most abundant chemical classes (based on the number of annotated compounds) included carboxylic acids and derivatives (356 compounds), fatty acyls (251), prenol lipids (229), and organooxygen compounds (217) (Figure 5a). PLS-DA of the metabolite profiles demonstrated clear separation among the treatment groups (Figure 5b). The CON samples formed a distinct cluster, separated from the LAB-inoculated treatments. ST, LP, and LS also showed tendencies to form sub-clusters, indicating treatment-specific metabolic signatures. The model exhibited good predictive ability (R2 = 0.948, Q2 = 0.755). Permutation tests (Figure 5c) confirmed the robustness of the PLS-DA model, with all permuted Q2 values being lower than the original Q2 value, and the Q2 regression line intercepting the *y*-axis below zero.

KEGG pathway analysis identified three major categories of differentially enriched metabolic pathways in the silage: Environmental Information Processing, Metabolism, and Genetic Information Processing (Figure 5d). Metabolism was the most significantly enriched category. Within the Metabolism category, biosynthesis of amino acids showed the greatest enrichment, followed by D-amino acid metabolism, arginine biosynthesis, phenylpropanoid biosynthesis, beta-Alanine metabolism, and pyrimidine metabolism.

### 3.4. Differential Metabolite Analysis

**Figure 6:**
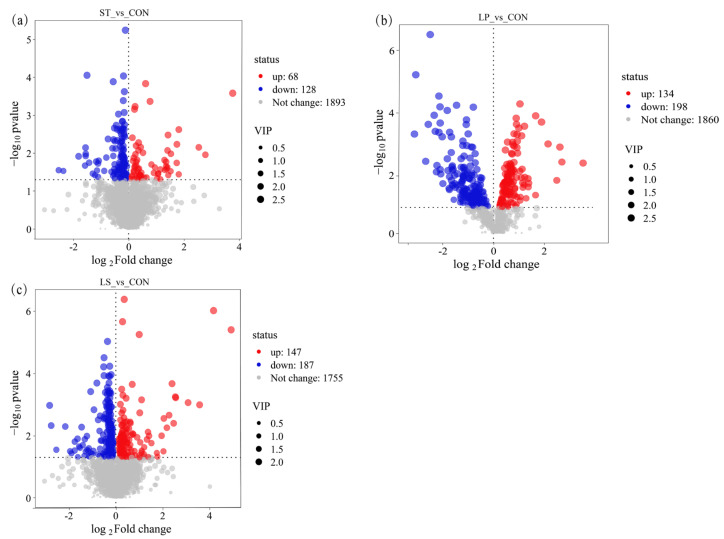
Volcano plot analysis of differential metabolites between treatment groups and the control group; volcano plots illustrating metabolic differences between (**a**) ST vs. CON, (**b**) LP vs. CON, and (**c**) LS vs. CON; the *x*-axis represents the log_2_ fold change (log_2_ FC), and the *y*-axis represents the −log_10_ significance of the *p*-value (−log_10_ *p*-value); CON, control group; ST, inoculation with *S. bovis*; LP, inoculation with *L. plantarum*; LS, co-inoculation with *S. bovis* and *L. plantarum*.

Differential metabolites between each inoculated treatment and the control were identified using the criteria VIP ≥ 1, fold change ≥ 2, and *p* < 0.05 (Figure 6). As shown in Figure 6a, the ST vs. CON comparison yielded 196 significantly differential metabolites in total, of which 128 were up-regulated and 68 were down-regulated in ST. The LP vs. CON comparison showed 332 differential metabolites (198 up, 134 down, Figure 6b), and the LS vs. CON comparison showed 334 (187 up, 147 down, Figure 6c).

According to KEGG annotations, the major classes of metabolites that changed in the inoculated silages (relative to CON) included carboxylic acids and derivatives, fatty acyls, benzene and substituted derivatives, organooxygen compounds, prenol lipids, glycerophospholipids, and flavonoids (Table S3). Many antioxidant-related metabolites in these classes were upregulated by inoculation. For example, several prenol lipids (e.g., humulenol I, digeranyl, deoxyneotigogenin) and flavonoids (e.g., glycyrrhizaflavonol A, hexahydroxyflavanone, malvidin-3-rutinoside, tricin) were present at higher levels in all LAB-inoculated silages compared to CON. Notably, the LS treatment upregulated a greater number of metabolites than either single-inoculant treatment, suggesting a more pronounced alteration of the silage metabolome with the combined inoculant.

**Figure 7:**
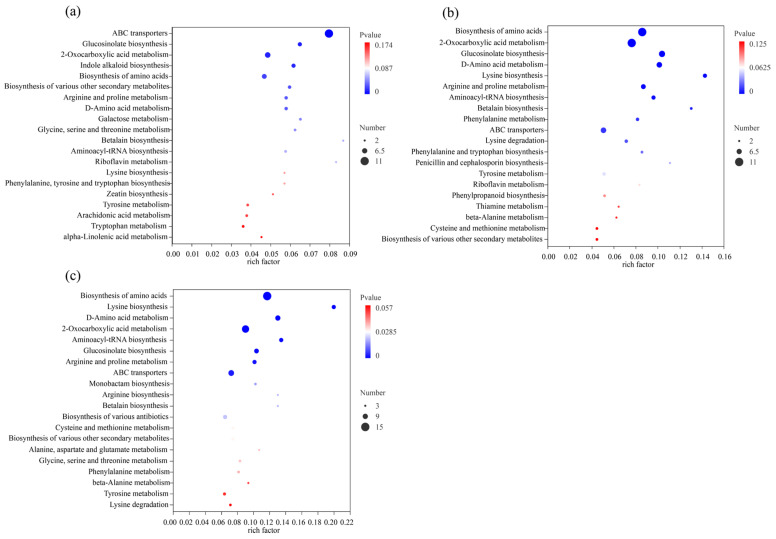
KEGG pathway enrichment analysis of differentially abundant metabolites. The analysis highlights enriched pathways resulting from comparisons between the control (CON) and inoculated groups: (**a**) *S. bovis* (ST), (**b**) *L. plantarum* (LP), and (**c**) co-inoculation (LS).

KEGG pathway enrichment analysis was performed on the differential metabolites for each comparison (Figure 7). The metabolites differing between ST and CON were significantly enriched in pathways such as ABC transporters, glucosinolate biosynthesis, 2-oxocarboxylic acid metabolism, indole alkaloid biosynthesis, and amino acid biosynthesis (Figure 7a). The differential metabolites between LP and CON were mainly enriched in pathways related to amino acid biosynthesis, 2-oxocarboxylic acid metabolism, glucosinolate biosynthesis, D-amino acid metabolism, and lysine biosynthesis (Figure 7b). The metabolites that differed between LS and CON were enriched in amino acid biosynthesis, lysine biosynthesis, D-amino acid metabolism, 2-oxocarboxylic acid metabolism, and aminoacyl-tRNA biosynthesis pathways (Figure 7c). Many of these pathways are central to carbon and nitrogen metabolism, highlighting that LAB inoculation primarily affected metabolic routes involved in energy production and amino acid turnover, which are crucial for silage fermentation quality.

### 3.5. Association Between the Bacterial Community and Metabolic Profile

**Figure 8:**
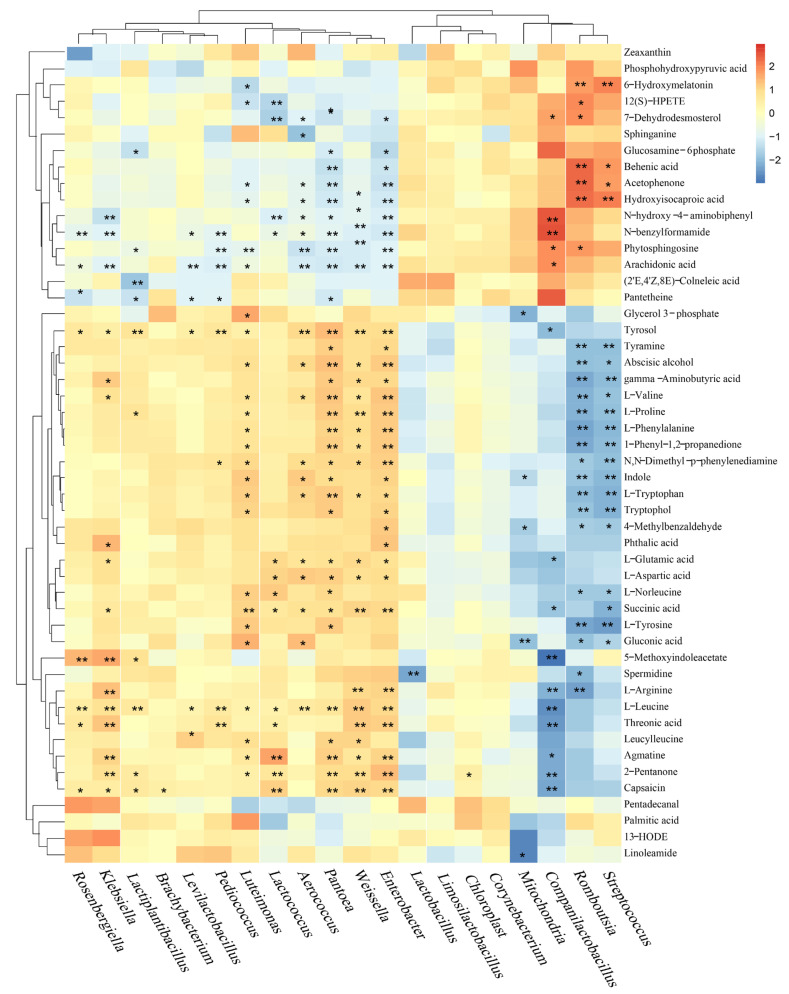
Construction of a correlation heatmap between different bacterial genera and metabolites based on Spearman analysis; color intensity indicates the strength of Spearman correlation, with red representing positive correlations and blue representing negative correlations; *with the indicate significant differences (*p < 0.05, **p < 0.01).

Spearman correlation analysis was conducted to explore relationships between dominant bacterial genera and metabolites (Figure 8). Genera typically considered beneficial LABs, such as *Streptococcus* and *Companilactobacillus*, generally showed negative correlations with numerous free amino acids (e.g., L-leucine, L-tyrosine, L-proline) and compounds like gluconic acid and indole. In contrast, genera often associated with spoilage or less optimal fermentation, including *Enterobacter*, *Weissella*, and *Pantoea*, exhibited positive correlations with several amino acids (e.g., L-valine, L-proline, L-phenylalanine) and were negatively correlated with metabolites like hydroxyisocaproic acid and N-benzylformamide. This inverse correlation pattern for most free amino acids—negative with predominant LAB and positive with potential spoilage genera—suggests that LAB-dominated fermentation limited proteolysis, while the presence of other bacteria was associated with higher levels of protein degradation products. *Romboutsia*, which was enriched in LS at day 30, displayed a correlation profile largely similar to beneficial LAB, being negatively correlated with many amino acids.

## 4. Discussion

This study investigated the effects of *S. bovis* (ST), *L. plantarum* (LP), and their combination (LS) on the fermentation quality, bacterial community dynamics, and metabolite profiles of dough-stage triticale silage. Our findings demonstrate that inoculation, particularly with the LS combination, significantly improved dough-stage triticale silage fermentation by accelerating acidification, modulating the bacterial succession towards beneficial LAB, and enriching for favorable metabolites while suppressing undesirable ones.

### 4.1. Inoculants Enhanced Fermentation Quality and Nutrient Preservation

Effective silage fermentation aims to rapidly reduce pH through organic acid production, primarily LA, thereby inhibiting spoilage microorganisms and preserving nutrients [7,28]. In this study, all inoculated treatments, especially LS, achieved lower pH and higher LA concentrations compared to the CON, consistent with previous reports on LAB inoculation [9,10]. The LS treatment yielded the highest LA (100.13 g/kg DM) and AA (27.61 g/kg DM) levels and the lowest pH (3.96) by day 30, indicating robust and efficient fermentation. This level of acidification is comparable to the results of Li et al. [10], who also achieved a pH below 4.0 in triticale silage using a *L. plantarum* and *Bacillus coagulans* combination, confirming that co-inoculation is a highly effective strategy for high-DM cereal forages. This superior performance of the combined inoculant likely stems from synergistic interactions. *S. bovis* is known for its rapid growth and acid production capabilities, potentially creating an initially favorable acidic environment that facilitates the subsequent activity of *L. plantarum* and other indigenous LAB [12,13,15]. This “kick-starting” effect by *S. bovis* could explain the faster starch and WSC consumption and LA accumulation observed in ST and LS treatments at day 7. The sustained activity, likely involving *L. plantarum* which is a strong homofermentative acid producer, then leads to the more profound acidification seen in LS by day 30. This aligns with findings that multi-strain inoculants can offer complementary benefits over single strains [11,29]. Notably, the observed rapid decrease in starch content and increase in LA in the ST and LS treatments at day 7 may be attributed to the amylolytic activity of *S. bovis*, as highlighted in our introduction [12,13,14]. This rapid substrate mobilization likely provided readily available fermentable sugars, fueling its fast proliferation and acid production, thereby giving these treatments an early fermentation advantage.

WSC are the primary substrates for LAB fermentation [30]. The more rapid and extensive utilization of WSC in inoculated silages, particularly LS, directly correlated with increased LA production. Conversely, the CON group retained higher WSC (32.78 vs. 20.72–29.07 g/kg DM) and starch contents (173.66 vs. 152.56–162.31 g/kg DM), indicating a less efficient fermentation. Reduced DML in all inoculated silages further supports their enhanced preservative efficacy, as DML reflects losses of organic matter through undesirable microbial activities or gaseous emissions [31].

Protein degradation into AN and other non-protein nitrogenous compounds is a critical factor affecting silage quality [32]. While AN concentrations were generally low across all treatments by day 30, indicating limited detrimental proteolysis, an interesting observation was the higher AN in the LS group (0.57–1.09 g/kg DM) throughout the ensiling. Concurrently, CP content was better preserved in *S. bovis*-inoculated silages (ST and LS) at day 7 (CP tends to be higher or loss is less). This seemingly contradictory result—higher AN yet better CP preservation—might reflect an accelerated initial phase of fermentation driven by *S. bovis*. The rapid microbial activity could lead to an initial burst of deamination from readily available amino acids (increasing AN), but the swift pH drop subsequently inhibits broader plant and microbial protease activity, thereby preserving more of the total protein from extensive breakdown [16,33,34,35]. The absence of BA in all samples is a positive indicator, suggesting clostridial activity was effectively suppressed, likely due to the rapid acidification and relatively high DM content of the triticale [28].

### 4.2. Inoculants Modulated Bacterial Community Succession Towards Beneficial LAB

Successful silage fermentation relies on a desirable microbial succession, where acid-tolerant LAB rapidly dominates and suppresses spoilage microorganisms [30,36]. Our 16S rRNA sequencing results demonstrate that LAB inoculation guided this process effectively. The significantly lower Shannon diversity in inoculated silages at day 7, compared to CON, indicates that the added LAB rapidly established dominance, creating a selective environment that limited the growth of a broader range of epiphytic microbes [36,37]. This is a hallmark of controlled fermentation, where a few highly efficient LAB (primarily Firmicutes) outcompete other bacteria, including many potentially detrimental Proteobacteria.

At day 7, *Lactiplantibacillus* and *Weissella* were the dominant genera, which is typical for the early stages of silage fermentation [32]. *Weissella* spp. are often active early due to their tolerance to moderate acidity and ability to utilize various sugars, though they are generally less acid-tolerant than *lactobacilli* and their dominance is often transient [38]. The higher abundance of *Lactiplantibacillus* in ST and LS at day 7 (44.88 and 43.00%, respectively), likely including the inoculated *L. plantarum* in LP and LS, and potentially stimulated indigenous *Lactiplantibacillus* by *S. bovis* activity in ST, underscores the immediate impact of inoculation. The inoculated *S. bovis* itself, taxonomically classified under *Streptococcus*, was also detected at higher levels in ST and LS treatments at day 7.

A key finding was the shift in dominance by day 30, where *Companilactobacillus* emerged as the most abundant genus in all inoculated silages (35.17–42.15%), particularly in LP and LS, while its levels were much lower in CON. This succession, where *Lactiplantibacillus* and *Weissella* declined and *Companilactobacillus* increased, suggests that *Companilactobacillus* species are well-adapted to the highly acidic, substrate-depleted conditions of later-stage silage [39]. This successional pattern, from early fast-growing heterofermenters (*Weissella*) and robust homofermenters (*Lactiplantibacillus*) to highly acid-tolerant specialists, is a known feature of well-controlled fermentations. For example, Yang et al. [38] observed a similar decline of *Weissella* as pH dropped in alfalfa silage. The emergence of *Companilactobacillus* as the terminal dominant genus, however, is a more novel observation in triticale silage, suggesting its specific fitness for the low-pH, high-starch environment. While not as extensively studied in silage as *Lactiplantibacillus* or *Limosilactobacillus*, some *Companilactobacillus* species are known for their extreme acid tolerance and have been associated with beneficial roles in other fermented foods or as probiotics [39,40,41]. Their prevalence in the well-fermented inoculated silages here suggests a significant, potentially positive, role in maintaining silage stability during prolonged storage. Suppressing undesirable genera such as *Rosenbergiella*, *Pantoea*, *Enterobacter*, and *Klebsiella* in inoculated silages is crucial, particularly by day 30. These Proteobacteria are often linked to aerobic spoilage, nutrient loss, and production of undesirable compounds [10,42]. Their reduction confirms the efficacy of LAB inoculation in creating an inhospitable environment for such detrimental microbes, primarily through rapid and sustained acidification.

### 4.3. Inoculants Reshaped Silage Metabolome, Enriching for Beneficial Compounds

The metabolomic analysis provided a deeper understanding of the biochemical changes driven by microbial activity [18,19]. The clear separation of metabolite profiles between CON and inoculated groups, and even among the different inoculant treatments, mirrored the distinctions observed in microbial communities, underscoring the strong link between microbiota and metabolome. The higher number of total detected metabolites compared to some other silage studies (e.g., rice straw [43], woody plants [44]) might be attributed to the specific substrate (triticale) and its relatively higher moisture content, which can foster greater microbial metabolic activity [45].

Consistent with fermentation data, carboxylic acids, particularly LA, were significantly elevated in inoculated silages, with LS showing the most pronounced increase. This enhanced acidogenesis is fundamental to the preservative action of LAB [46]. Beyond major organic acids, the upregulation of other potentially beneficial carboxylic acids and derivatives in inoculated silages, such as 3-hydroxyphenylacetic acid and indolelactic acid (especially in LP and LS), is noteworthy. 3-Hydroxyphenylacetic acid has reported antibacterial properties [47], while indolelactic acid, a tryptophan metabolite often produced by *Lactiplantibacillus* species, is known for its anti-inflammatory and gut-barrier enhancing effects [48]. The increased abundance of these compounds suggests that LAB inoculation, particularly with *L. plantarum*, may enhance the functional value of triticale silage beyond simple preservation.

A significant and desirable outcome of LAB inoculation was the general downregulation of free amino acids in ST, LP, and LS silages compared to CON. Excessive proteolysis during ensiling reduces the protein content and thus the nutritional value of silage [35,49]. The rapid acidification achieved by LAB inoculation inhibits plant proteases and proteolytic spoilage bacteria, thereby limiting the accumulation of free amino acids [33,34,50]. This result is highly consistent with metabolomic studies on other forages. For instance, Xu et al. [18] also reported a significant decrease in most free amino acids in sainfoin silage inoculated with L. plantarum, directly linking this to the inhibition of undesirable proteolytic bacteria. Our findings, combined with the AN data, provide strong metabolomic evidence for improved protein preservation in inoculated triticale silage. This was further supported by the correlation analysis, where LAB genera were negatively correlated with most amino acids, while spoilage-associated genera showed positive correlations. Flavonoids, plant secondary metabolites with antioxidant properties, were found at higher levels in all inoculated silages. This suggests an enhanced antioxidative capacity, which could protect silage from oxidative deterioration during storage and feed-out, and potentially offer health benefits to animals consuming the silage [19,51]. The upregulation of these compounds by LAB could be due to direct production, biotransformation of precursors, or better preservation of plant-derived flavonoids under improved fermentation conditions. KEGG pathway analysis indicated that LAB inoculation primarily influenced the extent and direction of existing metabolic pathways rather than introducing entirely novel ones. The enrichment of pathways related to amino acid metabolism (reflecting reduced proteolysis products), biosynthesis of various secondary metabolites, and carbohydrate metabolism in inoculated groups highlights the profound impact of the inoculants on the overall biochemical conversions during ensiling. Notably, the ‘starch and sucrose metabolism’ pathway was not significantly enriched in day-30 metabolomic analysis, despite clear evidence of starch utilization by day 7. This may contribute to the temporal dynamics of ensiling; the primary degradation of starch, which may be driven by *S. bovis*, is a rapid process occurring early in fermentation. By day 30, the metabolic flux through this pathway had likely subsided, with its intermediates (e.g., glucose) having been fully converted to organic acids. Consequently, the day-30 metabolic snapshot captures the accumulated end-products rather than the transient intermediates of early-stage catabolism. Future metabolomic studies at earlier time points (e.g., day 3 or 7) are therefore warranted to directly capture the signatures of active starch hydrolysis.

### 4.4. Interplay Between Microbial Community and Metabolites

The correlation analysis provided valuable insights into specific microbe–metabolite relationships. The strong negative correlation between beneficial LAB (e.g., *Streptococcus*, *Companilactobacillus*, *Limosilactobacillus*) and free amino acids, contrasted with the positive correlation for spoilage-associated genera (*Enterobacter*, *Pantoea*), robustly supports the role of LAB in limiting proteolysis. This is consistent with findings in other studies [43].

The positive correlation of *Lactiplantibacillus* with indole and tryptophan metabolites, including indolelactic acid, aligns with its known capacity to produce such compounds [48], suggesting a direct contribution to the enrichment of these potentially health-promoting metabolites in LP and LS silages. The accumulation of succinic acid, observed to be positively correlated with *Lactococcus* and *Enterobacter*, is another interesting finding. *Lactococcus* can produce succinate, and *Enterobacter* can also synthesize it [52,53,54]. Succinate can be converted to propionate in the rumen, a key glucogenic precursor for ruminants [54], thus its enrichment could be beneficial.

The genus *Romboutsia*, enriched in LS silage at day 30, showed a correlation profile similar to beneficial LAB (negative with amino acids, positive with some potentially beneficial compounds). While its role in silage is not well-defined, with some reports suggesting it may not favor active lactic fermentation [55,56], others indicate capabilities for complex carbohydrate degradation and SCFA production [57]. Its profile here warrants further investigation into its potential synergistic or beneficial role in the later stages of triticale silage fermentation. While this study provides comprehensive insights, some limitations should be acknowledged. Although metabolomics revealed many compounds, the functional impact of many less-characterized metabolites in silage or on animal health remains to be elucidated. Furthermore, this study focused on in-silo fermentation; evaluating the aerobic stability of these silages and their effects on animal performance would be valuable next steps. In summary, our findings offer a tangible solution for improving the fermentation of challenging high-starch forages. This leads to enhanced nutrient preservation, which in turn increases the feed’s economic value for the livestock industry. Furthermore, by reducing spoilage, our method ensures a more stable and higher-quality feed supply. This research provides a scientific basis for developing advanced, multifunctional silage inoculants for industrial applications.

## 5. Conclusions

Co-inoculation of *S. bovis* and *L. plantarum* (LS) significantly improves dough-stage triticale silage quality, outperforming single-strain inoculation. LS reduced pH most effectively and increased LA and AA concentrations via rapid starch breakdown. It fostered early *Lactiplantibacillus* and *Pediococcus* dominance, shifting to *Companilactobacillus*, while suppressing *Enterobacter* and *Pantoea*. Metabolomic analysis showed enriched organic acids and flavonoids with reduced proteolysis, enhancing nutrient retention. LS is a promising strategy for high-starch silage, with the potential for broader forage applications.

## Figures and Tables

**Table 1:** Chemical composition and microbial counts of fresh dough-stage triticale before ensiling.

Items	Content
DM, g/kg of FM	410
WSC, g/kg of DM	72.5
Starch, g/kg of DM	213
CP, g/kg of DM	85.7
NDF, g/kg of DM	632
ADF, g/kg of DM	301
pH	6.23
Microorganism (log_10_ CFU/g of FM)	
Lactic acid bacteria	3.22
Yeast	4.50
Mold	4.34
Aerobic bacteria	7.66

FM, fresh matter; DM dry matter; CP crude protein; NDF neutral detergent fiber; ADF acid detergent fiber; WSC water-soluble carbohydrate; values are presented as the mean of five replicate measurements (*n* = 5). The standard deviations were consistently low (less than 2% of the mean) and are therefore omitted for clarity of the baseline characteristics.

## Data Availability

The original contributions presented in this study are included in the article/Supplementary Materials. Further inquiries can be directed to the corresponding authors.

## Supplementary Materials

The following supporting information can be downloaded at: https://www.mdpi.com/article/10.3390/microorganisms13081723/s1. Figure S1: Number of LAB; Table S1: Microbial composition of triticale silage at the phylum level; Table S2: Microbial composition of triticale silage at the genus level; Table S3: Quantitative results of metabolites and annotation information.
