# Supplementary material for: Microbial and Metabolomic Insights into Lactic Acid Bacteria Co-Inoculation for Dough-Stage Triticale Fermentation
Source: Microorganisms. 2025 Jul 23;13(8):1723. doi: 10.3390/microorganisms13081723 (PMC12388346; doi:10.3390/microorganisms13081723)
Supplement: Supplementary file 1 [file microorganisms-13-01723-s001.zip › Table S1.pdf]

Table S1 Microbial composition of triticale silage at the phylum level %

Days	Treatments	Items					
		CON	ST	LP	LS	SEM	*P*-value
7 d	*Firmicutes*	74.94b	90.95a	89.54a	88.34a	2.48	< 0.001
	Proteobacteria	24.49a	8.72b	10.06b	11.18b	2.44	< 0.001
	Cyanobacteria	0.189	0.112	0.080	0.095	0.061	0.269
	Actinobacteria	0.348	0.156	0.266	0.295	0.115	0.363
	Bacteroidetes	0.010	0.026	0.043	0.078	0.045	0.394
	Others	0.028	0.031	0.012	0.018	0.014	0.290
30 d	*Firmicutes*	89.78b	93.62a	94.82a	92.20a	1.84	0.076
	Proteobacteria	9.70a	4.60b	4.21b	5.41b	1.18	0.001
	Cyanobacteria	0.314	0.311	0.371	0.174	0.123	0.454
	Actinobacteria	0.153	0.205	0.430	0.519	0.202	0.250
	Bacteroidetes	0.012	1.00	0.086	1.09	0.688	0.276
	Others	0.026b	0.249ab	0.079b	0.592a	0.209	0.064
