# Supplementary material for: Microbial and Metabolomic Insights into Lactic Acid Bacteria Co-Inoculation for Dough-Stage Triticale Fermentation
Source: Microorganisms. 2025 Jul 23;13(8):1723. doi: 10.3390/microorganisms13081723 (PMC12388346; doi:10.3390/microorganisms13081723)
Supplement: Supplementary file 1 [file microorganisms-13-01723-s001.zip › Table S2.pdf]

S2 Microbial Composition of Triticale Silage at the Genus Level %

Days	Treatments	Items					
		CON	ST	LP	LS	SEM	*P*-value
	Lactiplantibacillus	33.34bc	44.88a	38.19b	43.00a	2.61	0.002
	Weissella	28.19b	29.67ab	35.08a	31.13ab	2.95	0.152
	Companilactobacillus	1.11b	1.49b	1.53b	5.11a	0.789	< 0.001
	Rosenbergiella	10.81a	1.14b	4.39b	1.40b	1.91	< 0.001
	Levilactobacillus	4.73ab	7.36a	5.33ab	3.52b	1.18	0.035
	Limosilactobacillus	0.747	1.97	0.997	0.964	4.38	0.152
	Chloroplast	0.189	0.111	0.079	0.094	0.057	0.269
	Mitochondria	0.190	0.103	0.047	0.16	0.114	0.617
	Lactobacillus	0.064	0.061	0.392	0.053	0.212	0.331
7 d	*Pantoea*	1.94a	0.537b	0.835ab	0.727b	0.538	0.076
	Pediococcus	0.702b	3.03a	2.89a	2.34a	0.584	0.004
	Enterobacter	1.96a	1.04b	0.806c	1.44bc	0.210	< 0.001
	Klebsiella	1.67a	1.25a	0.647b	1.66a	0.248	0.002
	Aerococcus	0.122	0.231	0.316	0.391	0.162	0.414
	Luteimonas	0.009	0.003	0.003	0.007	0.005	0.615
	Brachybacterium	0.090	0.049	0.102	0.089	0.039	0.578
	Lactococcus	0.159b	0.351a	0.138b	0.377a	0.050	< 0.001
	Streptococcus	0b	1.25a	0b	0.728a	0.265	< 0.001
	Corynebacterium	0.055	0.017	0.067	0.059	0.033	0.480
	Romboutsia	0.010b	0.013b	0.018ab	0.030a	0.006	0.047
	Others	9.02a	5.38bc	3.98c	6.67b	0.861	< 0.001
30 d	*Lactiplantibacillus*	25.23a	16.17b	22.01ab	18.44b	3.76	0.132

Weissella	28.48a	20.09b	19.50b	17.24b	4.42	0.001
Companilactobacillus	21.13b	35.17ab	42.15a	35.80ab	11.78	0.041
Rosenbergiella	1.98	1.09	0.842	1.18	0.511	0.178
Levilactobacillus	5.07	3.75	3.10	4.19	0.972	0.269
Limosilactobacillus	2.88ab	13.03a	3.24ab	1.76b	4.69	0.097
Chloroplast	0.369	0.311	0.314	0.174	0.123	0.465
Mitochondria	0.458	0.175	0.104	0.235	0.211	0.398
Lactobacillus	0.640	1.11	3.43	8.37	5.73	0.532
Pantoea	0.985a	0.114b	0.217b	0.174	0.257	0.012
Pediococcus	1.25	1.11	0.927	0.904	0.207	0.319
Enterobacter	1.01a	0.473b	0.406b	0.317b	0.125	< 0.001
Klebsiella	0.988a	0.478b	0.320b	0.363b	0.140	0.001
Aerococcus	0.097a	0.034b	0.042b	0.044b	0.024	0.078
Luteimonas	0.001	0	0.006	0	0.003	0.187
Brachybacterium	0.015	0.02	0.028	0.014	0.016	0.826
Lactococcus	0.464a	0.181b	0.190b	0.202b	0.086	0.012
Streptococcus	0.002b	0.090a	0.003b	0.088a	0.030	0.023
Corynebacterium	0.183	0.027	0.028	0.281	0.163	0.352
Romboutsia	0.114c	0.501ab	0.310b	0.715a	0.147	0.006
Others	5.37	6.04	6.00	9.46	1.93	0.181
