# Supplementary figures and images for: Microbial and Metabolomic Insights into Lactic Acid Bacteria Co-Inoculation for Dough-Stage Triticale Fermentation
Source: Microorganisms. 2025 Jul 23;13(8):1723. doi: 10.3390/microorganisms13081723 (PMC12388346; doi:10.3390/microorganisms13081723)

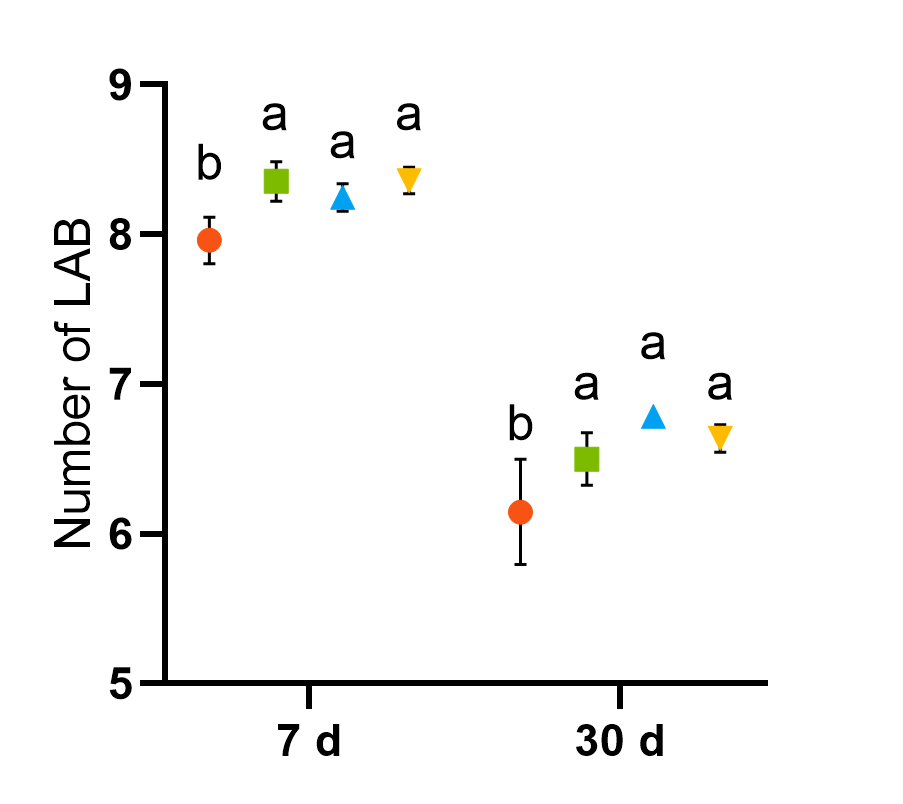

Supplement: Supplementary file 1 [file microorganisms-13-01723-s001.zip › Fig. S1.tif]
